# Development of Parkinsonism in a Patient with Central Pontine Myelinolysis

**DOI:** 10.3390/neurolint14030055

**Published:** 2022-08-25

**Authors:** Annibale Antonioni, Vittorio Rispoli, Patrik Fazio, Nico Golfrè Andreasi, Vittorio Govoni, Enrico Granieri

**Affiliations:** 1 Unit of Clinical Neurology, Department of Neuroscience and Rehabilitation, University of Ferrara, 44 121 Ferrara, Italy; 2 Department of Clinical Neuroscience, Karolinska Institutet, 171 77 Stockholm, Sweden

**Keywords:** central pontine myelinolysis, parkinsonism, osmotic demyelination syndrome, ODS, movement disorder

## Abstract

Osmotic demyelination syndrome (ODS) is caused by damage to the pons myelin sheath and nerve cells. Although the pathophysiological mechanism responsible for the damage is not yet fully understood, it is currently believed that osmotic-type changes (especially if they are massive and too rapid) cause oedema that leads to compression and, subsequently, demyelination of white matter fibres. It generally manifests with acute paraparesis/tetraparesis, dysphagia, dysarthria, diplopia, and loss of consciousness, as well as hallucinations, spasms, and other neurological symptoms related to brainstem damage. In extreme cases, the locked-in syndrome may also appear. Of note, in some cases an association between osmotic demyelinating damage and the onset of movement disorders has been documented and, although the pathophysiology is still unknown, a correlation has been postulated between ODS and movement disorders. Here, we present a patient with ODS who developed parkinsonism, thus supporting the hypothesis of a correlation between these pathological events.

The term osmotic demyelination syndrome (ODS) encompasses disorders characterised by myelin disruption in various encephalic regions and, currently, it comprises central pontine myelinolysis (CPM) and extra-pontine myelinolysis (EPM) [1]; after the first report by Adams and Victor (1959) [2], who described patients with myelinolysis of pontine fibres, many other reports were published postulating that other regions of the brain could be involved with the possible development of movement disorders. Although it can arise asymptomatically, it often results in acute encephalopathy, flaccid or spastic tetraparesis, brainstem disorders, death, and movement disorders [3]. In fact, some cases of ODS associated with chorea, dystonia, ataxia, and catatonia have been observed, although the evidence is still limited [3]. These findings were summarised in a review [4,5]. Although its pathophysiology is not fully understood, among the best-known risk factors are chronic alcoholism, liver transplantation, rapid and massive correction of hyponatremia, malnutrition, and hypokalaemia [3]. Among the disorders associated with ODS, parkinsonism, i.e., a clinical picture characterised by rest tremor, rigidity, bradykinesia, and balance disturbances, can also occur in rare cases. This condition is clinically similar to Parkinson’s disease (PD), but is provoked by numerous and variable causes, whereas PD is a progressive neurodegenerative disease [5].

We aim to update the description of a patient monitored at the Neurology Department of Ferrara University that was published in 2010 [6]. He was a 69-year-old Caucasian male, with a long personal history of type II diabetes, hypertension, hypercholesterolemia, peripheral arterial obstruction, and depression (with a component of hypochondria) who worked in contact with glues and cement for about 40 years. The family history reported no neurological diseases. In 2007, the patient was referred to our centre complaining about severe headaches described as a “daily and increasingly strange sensation of pain in the head”, which was present every day and which had increased over time (started about 10 years earlier). The pharmacological medical history showed the use of clomipramine (for about 10 years), lorazepam, fluoxetine, delorazepam, oxazepam, and olanzapine (for about 1 year). Cognitive function, cranial nerve, and upper and lower limb functions were normal on neurological examination. Brain MRI disclosed a lesion in the pons compatible with myelinolysis. The actual reason why he presented this lesion was not clear, but in the case report, we discussed the possible role in the etiopathogenesis of his chronic use of anti-depressive drugs and the exposure to glue and chemical agents for 40 years [6]. Moreover, MRI showed signs of vascular encephalopathy, probably associated with his long history of hypertension and type II diabetes. After 4 years, at the age of 71, asymmetric signs of parkinsonism (rigidity and bradykinesia in the left hemibody) were found during clinical evaluation, so therapy with levodopa/carbidopa was started and the symptoms improved. A DaT-Scan SPECT revealed a moderate asymmetric reduction of pre-synaptic dopamine transporters in the right striatum (Figure 1).

In the years to follow, the symptoms slowly worsened, and at the last evaluation in 2019, during an ON state, the neurological assessment showed a hypomimic patient with stuttering and mild–moderate bilateral bradykinesia and rigidity, both more prominent in the left hemibody. No rest tremor was detected. The only way he was able to stand up from a chair was with the help of his arms; he had a moderate stooped posture without postural reflex impairment and his gait was asymmetric (his left foot did not clear the floor while walking). The patient was still independent in activities of daily living (ADLs, score 6/6) and almost independent in instrumental activities of daily living (IADLs, score 7/8). The Mini Mental State Examination (MMSE) score was 24.7. He also complained about urinary urgency. Of note, the patient had no sensory disturbances, either subjective or objective. Anosmia, carefully investigated by means of the Sniffin’ Sticks test (Burghardt^®^, Pinneberg, Germany), was absent. Moreover, a polysomnographic investigation performed to check for REM sleep behavioural disorder (RBD) or other sleep disturbances ruled out such problems, and the patient did not complain of daytime sleepiness. Finally, myocardial scintigraphy (123I-MIBG) was normal, documenting the absence of cardiac adrenergic denervation. We managed to obtain satisfactory motor symptom control with a maximum dose of levodopa/carbidopa of 150 mg tid.

Movement disorders as a consequence of ODS have not been described in many case reports; in several of the patients examined, MRI could detect a lesion compatible with myelinolysis in various sites of the basal ganglia, such as the striatum and even in substantia nigra [5]. In other patients, however, cerebral lesions compatible with myelinolysis were only evident in the pons, and they still had movement disorders, with parkinsonism as the most common manifestation. Symptoms usually manifest acutely, but also late presentations with a chronic and progressive course have been described, both for dystonia and parkinsonism [5,7]. We were not able to precisely date the myelinolysis of the pons in our patient, as the only symptom he manifested was an intense daily headache for many years before he underwent a first MRI, but we can affirm that the onset of parkinsonism was surely delayed with respect to the pontine lesion compatible with myelinolysis.

We cannot entirely exclude a neurodegenerative origin of the parkinsonian features observed or vascular parkinsonism. The latter is not consistent with our findings because of the slow progression of the same syndrome which is asymmetric parkinsonism without additional signs. Moreover, the lesions in deep nuclei remained stable over time (Figure 2). ODS and idiopathic Parkinson’s disease (PD) could be independently present in our patient, but it has been postulated that pontine myelinolysis may act as a trigger of a neurodegenerative process [7]; this may be the case for our patient. Of note, the patient had no other non-motor symptoms typical of PD, i.e., sleep disturbances and sensory disturbances, such as reduced olfactory perception, a typical and early symptom of this disease [8,9]. Furthermore, the 123I-MIBG documented findings are more likely compatible with parkinsonism than PD [10]. These data seem to make the hypothesis of a clinical case of parkinsonism, as opposed to one of PD, more plausible. Moreover, the late and progressive evolution could be linked to abnormal neuronal and synaptic network rearrangements associated with retrograde degeneration [5].

We have no knowledge of other cases of parkinsonism associated with an otherwise asymptomatic ODS reported in the literature so far. We believe that our findings are intriguing, and confirm that the connection between ODS and late-onset movement disorders is far from being comprehensively understood. However, this case report could represent further possible evidence to support the correlation (already hypothesised in the literature) between ODS and late-onset movement disorders.

## Figures and Tables

**Figure 1:**
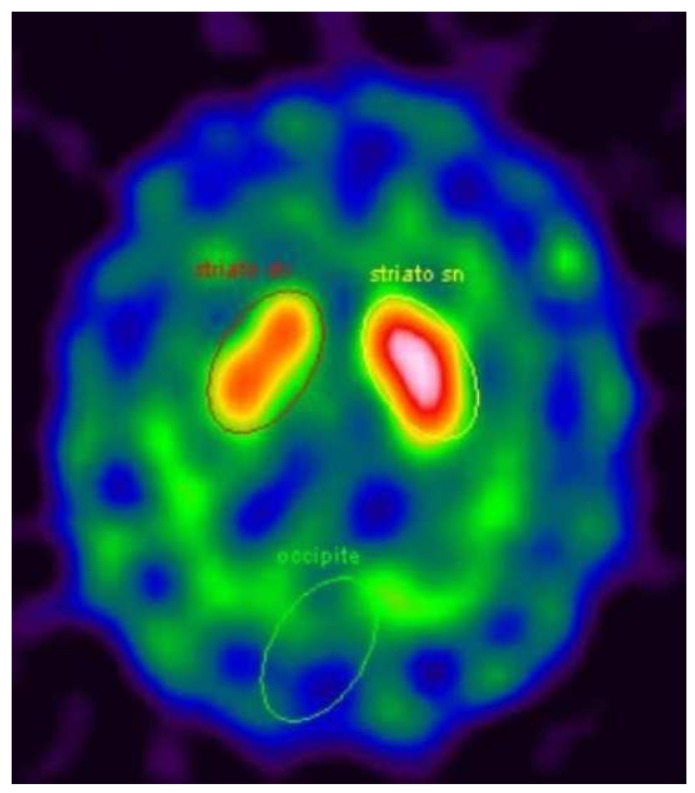
DaT-Scan SPECT showing asymmetric reduction of dopamine transporters in right striatum.

**Figure 2:**
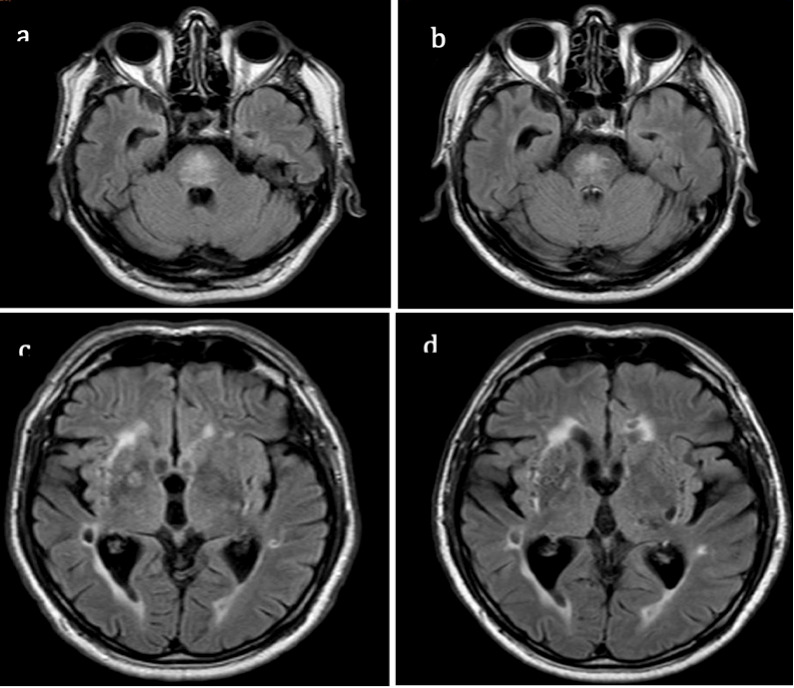
MRI fluid-attenuated inversion recovery (FLAIR) in 2007 (**a**,**c**) and in 2014 (**b**,**d**). During these years, there was a reduction in the volume of pontine myelinolysis (**a**,**b**); the hyperintensities in deep white matter and in deep nuclei are similar (**c**,**d**).

## Data Availability

Not applicable.
